# The Dark Side of Study: When Study Negatively Affects Relationships and School Climate. The Study-Relationships Conflict Scale

**DOI:** 10.5964/ejop.v15i2.1567

**Published:** 2019-06-07

**Authors:** Yura Loscalzo, Marco Giannini

**Affiliations:** a Department of Health Sciences, School of Psychology, University of Florence, Florence, Italy; Webster University Geneva, Geneva, Switzerland; University of Wroclaw, Wroclaw, Poland

**Keywords:** students’ wellbeing, study addiction, study engagement, studyholism, study obsession, work addiction, workaholism, work-family conflict

## Abstract

This study proposes a new instrument for evaluating the Study-Relationships Conflict, or the conflict that may exist between study and personal relationships with family, friends, schoolmates, and teachers. We recruited a sample of 598 Italian University students (age: M = 22.58 ± 3.85) of different majors. By means of Exploratory and Confirmatory Factor Analyses, we reduced the 16-item pilot version to nine items and three factors: 1) Quarrels at School—QS; 2) Relationship Impairment—RI; 3) Family and Friends’ Complaints—FFC. Moreover, we analyzed the correlation between these scales and some academic indicators: Grade Point Average (GPA) and time spent studying. The results showed that the Study-Relationships Conflict Scale (SRCS) has good psychometric properties. In addition, GPA positively correlates with the FFC scale; while time spent studying correlates positively with both the RI and the FFC scales. Finally, QS has a statistically and low significant positive correlation with the hours a day of study before exams. The SRCS will be useful in future research aiming to analyze how studying behaviors could affect social and school relationships. Moreover, it could also be used as a quick screening for detecting student at-risk of high social impairment due to their overstudying, and for developing preventive interventions.

Work and study are respectively the primary activity for workers and students. In our society, overworking/overstudying is usually seen as a good and valuable behavior. Though, when it becomes excessive, it could lead to clinical conditions such as Workaholism (or work addiction; Oates, 1971) and Study Addiction (Atroszko, Andreassen, Griffiths, & Pallesen, 2015) or Studyholism (or study obsession; Loscalzo & Giannini, 2017a).

In literature, a lot of attention has been devoted to Workaholism and to the adverse effects that could be associated with it at the individual, organization and family levels. For example, Workaholism could lead to psychological and physical impairment, to lower work performance, to higher aggressive workplace behaviors, and to work-family conflict (Andreassen, 2014; Clark, Michel, Zhdanova, Pui, & Baltes, 2016; Loscalzo & Giannini, 2017b; Sussman, 2012). In the same line, Atroszko et al. (2015) recently found that Study Addiction has adverse consequences for students. Moreover, in their theoretical model, Loscalzo and Giannini (2017a) suggested some negative outcomes that may be associated to Studyholism: physical and psychological health impairment, lower academic performance, aggressive behaviors at school/University and an impairment of the relationships with relatives, friends/partners, and classmates.

In their papers, Loscalzo and Giannini (2017a, 2018a, 2018b) stressed that Study Addiction and Studyholism are two different constructs due to their different theoretical frameworks. Study Addiction has been conceptualized referring to the core components of substance addictions (Atroskzo et al., 2015). Studyholism, instead, has been conceptualized going beyond the addiction model and, more specifically, as a problematic behavior which may be more similar to an obsession than to an addiction (Loscalzo & Giannini, 2017a, 2018a, 2018b, 2018c; Loscalzo, Giannini, & Golonka, 2018). Moreover, Loscalzo & Giannini (2017a) introduced the distinction between Engaged and Disengaged Studyholics, hence adopting the Heavy Work/Study Investment model (see Snir & Harpaz, 2012). However, they also pointed out that, besides the different operationalization of the constructs, Study Addiction and Studyholism are related to the same behavior, that is studying, or more specifically problematic overstudying. Therefore, the studies about Study Addiction and Studyholism are both critical as they might highlight the negative outcomes associated with problematic overstudying. Hence, Loscalzo and Giannini (2017a) suggested that future studies should analyze their hypothesized relationships between problematic overstudying and its negative consequences. Though, given that it is a new construct, many variables cannot currently be evaluated since there are not instruments for measuring such aspects. Among these variables, there is the Study-Relationships Conflict.

## The Study-Relationships Conflict

The Study-Relationships Conflict (SRC) is assumed to be similar to the construct of Work-Family Conflict (WFC), even if we have adapted it to the study and student’s life context.

As far as the WFC is concerned, it is defined as a source of stress for many individuals (Carlson, Kacmar, & Williams, 2000) and, more specifically, as an inter-role conflict between work and family domains, which may be expressed in three different forms: time-based, strain-based, and behavior-based (Greenhaus & Beutell, 1985). Moreover, as suggested by Greenhaus and Beutell (1985), the WFC may have two directions: the work interferes with family functioning (work-to-family conflict), or the family interferes with the work functioning (family-to-work conflict). In the workaholism literature, the Work-Family Conflict Scale (WFCS; Carlson et al., 2000) is one of the most used instruments for evaluating the WFC, both in its work-to-family and family-to-work conflict components. Though, the content of some of its items is not suitable to be adapted to the school context; moreover, the WFCS items do not address all the main students’ relationships besides family, such as those with friends and classmates. Finally, the WFCS does not address aggressive behaviors, as it focuses on the work-family conflict specifically.

Hence, we propose the theoretical construct of SRC, and an instrument for evaluating it, since it is conceptualized as a different construct than the WFC.

We define the SRC as an inter-role conflict between study and relationships, which includes both social (family, friends, and partner) and school (teachers and classmates) relationships. We specify that there may be both a Study-to-Relationships Conflict (S-R-C) and a Relationships-to-Study Conflict (R-S-C). The S-R-C is the form addressed by the instrument we present in this paper, as we are interested in the influence that overstudying may have on social and school relationships. However, it is crucial to analyze also the R-S-C as there might be students whose low grades or aggressive behaviors at school/University are due to problems they are experiencing within their family (e.g., parents’ divorce, disagrees within family, illness of a family member), with their friends/partners (e.g., quarrels with friends or partner, use or abuse of addictive drugs in social situations, social isolation), and teachers or classmates (e.g., low wellbeing at school). Finally, we also stress that the SRC might be evident in both a relationships impairment (e.g., losing some friends, not going out with friends) and in overt aggressive behaviors (e.g., quarrels with teachers due to bad grades).

It is important to have a construct and an instrument related to the conflict that may arise between the students’ main life domains, as the ones of students (pre-adolescents, adolescents, and youths) are different as compared to the ones of workers (adults). While for workers the two main life domains are work and family, for students these are study and social relationships, and the latter does not include the family only but also schoolmates, teachers, and friends.

There are indeed some differences in the social lives of students and workers. Besides the well-known changes in the biological and psychological areas that characterize pre-adolescence and adolescence (e.g., development of primary and secondary sexual characters, meta-cognitive abilities, and moral reasoning), there are also significant changes in the social domain. Havighurst (1952) pointed out that, among the developmental task/challenges of the adolescents, there are: building new and mature relationships with peers of both the genders, acquiring emotional independence from the family and other adults, and preparing themselves toward a job. In the same line, Erikson (1968) theorized that the developmental stage of adolescence (identity vs. role confusion) is characterized by social relationships with peers that culminate in a heterosexual relationship. Next, the youths experience the intimacy vs. isolation stage of development, whose main features are the social interaction with a person of opposite sex and the acceptance of the adults’ duties, also including the relationship with a partner. Finally, in the adult developmental stage, people have already built their main relationships, and they are more oriented to be productive.

It looks clear that there are some differences between the social relationships of adolescents and youths and those of adults. While students are involved in building new relationships, workers have a clear set of stable relationships, generally including a spouse and children.

In addition, it is usually assumed that peers begin to have a higher influence on adolescents, as compared to parents and other adults. During adolescence, there is an increase in the development of peer relationships, in both the friendship and sentimental levels. A good relationship with peers during this developmental stage is critical as it is an index of the adolescents’ psychological well-being and a protective factor against psychosocial problems (Hartup & Stevens, 1997). However, the family maintains an essential role in adolescence, especially when the adolescents face stressful situations such as mental health problems or physical illness (Hauser, Vieyra, Jacobson, & Werlieb, 1985). Hence, for students, both friends and family are equally important.

Moreover, while adolescents and youths mainly fulfill the roles of son/daughter, sibling, friend, and student, adults meet the roles of parent, spouse, and worker instead. It should also be noted that while the roles associated to adolescence and youth are mostly mandatory (with the exception of being a College student, which is a free choice), the roles associated to adulthood are instead generally due to a free choice, as an adult may decide if he/she wants to get married and have children, and he/she may also choose to give up a job.

Finally, there are different consequences related to a conflict between the two primary domains of a worker and a student. The WFC may be associated with adverse outcomes such as partner’s dissatisfaction, neglecting the duties toward the spouse and the children, higher distress in children, work dissatisfaction and turnover intention (e.g., Erdamar & Demirel, 2014; Fischer, Zvonkovic, Juergens, Engler, & Fredericj, 2015; Nohe & Sonntag, 2014; Vahedi, Krug, Fuller-Tyszkiewics, & Westrupp, 2018). The SRC may be instead mainly associated to an impairment of peer and family relationships, with a higher risk for developing psychological disorders (Hartup & Stevens, 1997). In addition, especially as far as the Relationships-to-Study Conflict is concerned, it may be associated to lower grades and higher risk for dropout, and hence to a low-level job later in life.

Given these features of social relationships in adolescence, as well as in the “late adolescence” that characterizes the youths still living within their family (such as, usually, College students), we posit that, when studying SRC, we should evaluate it in both the social (family, friends, and partner) and school (teachers and classmates) domains. Finally, we suggest that this conflict may be evident not only in the form of relationships’ impairment, but also in overt aggressive behaviors, such as quarrels with classmates, teachers, and family members due to school issues (e.g., low grades).

## The Present Study

The main aim of this paper is to propose a new instrument to be used for evaluating the Study-to-Relationships Conflict as defined in the present report or, more specifically, the negative impact of the study on social and school relationships, both in term of aggressiveness and social impairment. This instrument will be useful in future research aiming to address the impact of overstudying on personal relationships and school climate, but it could also be used as a quick screening of students with the aim of detecting the ones who are experiencing social impairment due to their studying behaviors. They could receive a preventive intervention designed for helping them to develop more positive and constructive attitudes toward studying, as to preserve their relationships.

We also analyzed the correlations between Study-Relationships Conflict (i.e., quarrels at school, relationship impairment, and family and friends’ complaints) and academic indicators, that is time investment in the study (both generally and before exams) and Grade Point Average (GPA). More specifically, we hypothesized that: 1) time investment in studying is not associated with aggressive behaviors at University, as we speculate that the students who spend a lot of time studying feel good in the school context, and hence they do not argue with teachers and schoolmates about grades or other school matters; 2) higher time investment in the study is associated to higher relationship impairment and family and friends’ complaints, since parents, siblings, and friends may negatively react to the reduced amount of time dedicated to them as compared to study (Adkins & Premeaux, 2012; Major, Klein, & Ehrhart, 2002); 3) a higher GPA is not associated with quarrels at University, as we posit that the students are satisfied with their grades, neither to relationship impairment with family and friends; 4) a higher GPA is associated with more complaints from family and friends, as we speculate that they might blame these students for being too much focused on studying.

## Method

### Participants

We got the participation of 598 University students aged between 18 and 56 years (*M* = 22.58 ± 3.85; 82.8% females, 17.2% males). The students attended University in many different Italian cities, with Florence being the most represented (20.2%), followed by Milan (10.9%). This total sample is made by a first sample of 290 students that we gathered to perform Exploratory Factor Analysis (EFA) and by a second sample of 308 students on which we did Confirmatory Factor Analyses (CFAs). Table 1 shows the characteristics of the samples.

**Table 1:** Socio-Demographic Characteristics of the Two Samples and Total Sample

Characteristic	Sample 1 (*n* = 290)	Sample 2 (*n* = 308)	Total Sample (*n* = 598)
Age (*M* ± *SD*)	22.41 ± 3.42	22.75 ± 4.22	22.58 ± 3.85
Gender (%)			
Females	83.1	82.5	82.8
Males	16.9	17.5	17.2
Major (%)			
Psychology	18.6	19.5	19.1
Medicine	16.9	17.5	17.2
Other	64.5	63.0	63.7
Year (%)			
1	18.3	16.9	17.6
2	20.0	20.1	20.1
3	26.6	23.1	24.7
4	15.5	17.8	16.6
5	19.7	22.1	20.9

### Materials

#### Study-Relationships Conflict Scale (SRCS)

We developed a pool of 16 items covering social (family, friends, and partner) and school (teachers and classmates) problematic relationships due to study (including aggressive behaviors towards teachers and classmates). Eight items address each of the two relationships’ domains. The participants have to fill out the questionnaire indicating how much they agree with each of the sentences using a 5-point Likert scale ranging between 1 (*Completely disagree*) and 5 (*Completely agree*). The participants of the first sample filled the 16-item pilot version of the SRCS, while the participants of the second sample filled the 9-item reduced version. We designed this scale both in the instructions and in the items using a language that could be easily understood by College students, but also by younger students, such as pre-adolescents and adolescents.

#### Academic Variables

The participants answered some questions related to their academic behaviors. More specifically, they reported approximately how many hours a day and how many days a week they spent studying, both generally and before exams. Moreover, we asked them to tell their Grade Point Average (GPA), as an academic performance indicator.

### Procedure

Once we got the authorization to conduct the study by the Department of Health Sciences of the University of Florence, we administered an online questionnaire including the Study-Relationships Conflict Scale and some demographic (i.e., age and gender) and study-related (e.g., GPA and hours a day of study generally) questions. In the questionnaire, we included a first page in which we presented the aims of the research and all the information about the anonymity and the right to stop filling the questionnaire at any time. At the end of this page, we highlighted that by keep on filling the questionnaire, the participants agreed to take part in the research and hence gave their informed consent.

### Data Analysis

We performed the analyses using SPSS.24 and AMOS.22. First, we conducted Exploratory Factor Analysis (EFA; Principal Axis Factoring and Promax Rotation) on the first sample of participants (*n* = 290), aiming to reduce the number of items of the Study-Relationships Conflict Scale (SRCS). After that, we cross-validated the factor structure of the scale through Confirmatory Factor Analyses (CFAs) on the second sample of students (*n* = 308). To evaluate the goodness of fit of the model, we made reference to the following indexes and cut-off scores: the χ^2^/*df* ratio, which indicates a good fit if it is less than 3 (Byrne, 2001); the Goodness of Fit Index (GFI), the Comparative Fit Index (CFI), and Tucker-Lewis Index (TLI), which have the same cut-off values: < 0.90 lack of fit, 0.90–0.95 good fit, > 0.95 excellent fit (Hu & Bentler, 1999); and the Root Mean Square Error of Approximation (RMSEA), whose cut-off values are: < 0.05 excellent fit, 0.05–0.08 acceptable fit (Reeve et al., 2007). We performed EFA as a first step, in order to reduce the number of items of the scale, because CFA “is primarily a method for assessing the construct validity of measures and it is not a means for data reduction unlike EFA” (Singh, Junnarkar, & Kaur, 2016, p. 27). Indeed, “EFA is normally the first step in building scales or a new metrics” (Yong & Pearce, 2013, p. 79), which applies in the present study as we developed a new instrument for evaluating a new construct and, in order to reach this aim, we created a pool of items to be reduced by means of EFA.

Finally, we evaluated the internal reliability (Cronbach’s alpha) and the item-total correlations of the three scales of the final version of the SRCS and their correlations (Pearson) with the academic indicators (i.e., GPA and hours of study a day and days of study a week generally and before exams) on the total sample (*n* = 598).

## Results

As a first step, we performed an Exploratory Factor Analysis (EFA; Principal Axis Factoring, Promax Rotation) on the first sample (*n =* 290), aiming to evaluate the factor structure and to obtain a shorter version of the 16-item pilot version of the SRCS.

Following the criterion of the eigenvalues greater than one and based on the scree-plot, we extracted three factors, which explain the 31.95%, the 50.05% and the 61.67% of the variance cumulatively. With reference to the content of the three items belonging to the same factor (for a total of nine items), we labeled the three factors: 1) Quarrels at School—QS, 2) Relationship Impairment—RI, and 3) Family and Friends’ Complaints – FFC (Table 2 shows the saturation values of the three factors).

**Table 2:** Exploratory Factor Analysis of the Study-Relationships Conflict Scale (SRCS), *n =* 290

SRCS Item [English translation]	QS	RI	FFC
1. Non sono molto presente in famiglia a causa dello studio. [*Family life is compromised by my studying.]*		.53	
2. Spesso litigo con i miei insegnanti. [*I often quarrel with my teachers.*]	.56		
3. I miei genitori e/o amici mi dicono che studio troppo. [*My parents and/or friends tell me that I study too much.*]			.47
4. Ho perso alcuni amici, a causa dello studio. [*I have lost friends, because of my studying.*]		.48	
5. Spesso rispondo male ai miei insegnanti e/o ai miei compagni. [*I often speak impolitely to my teachers and/or my classmates.*]	.79		
6. I miei amici mi considerano un/a secchione/a. [*My friends consider me a swot.*]			.88
7. Spesso litigo con i miei compagni. [*I often quarrel with my classmates.*]	.67		
8. A causa dello studio, non esco molto con i miei amici e/o con il/la mio/a fidanzato/a. [*Because of my studying, I do not go out much with my friends and/or my boyfriend/girlfriend.*]		.79	
9. I miei compagni mi prendono in giro perché penso solo allo studio. [*My classmates joke me because I only think about studying.*]			.44

*Note.* Principal Axis Factoring, Promax Rotation; Factor loadings below .30 are not presented. QS = Quarrels at School; RI = Relationship Impairment; FFC = Family and Friends’ Complaints. *If interested in using this scale, you can write to the first author for having the instruction to be used jointly to the scale itself.*

**Figure 1:**
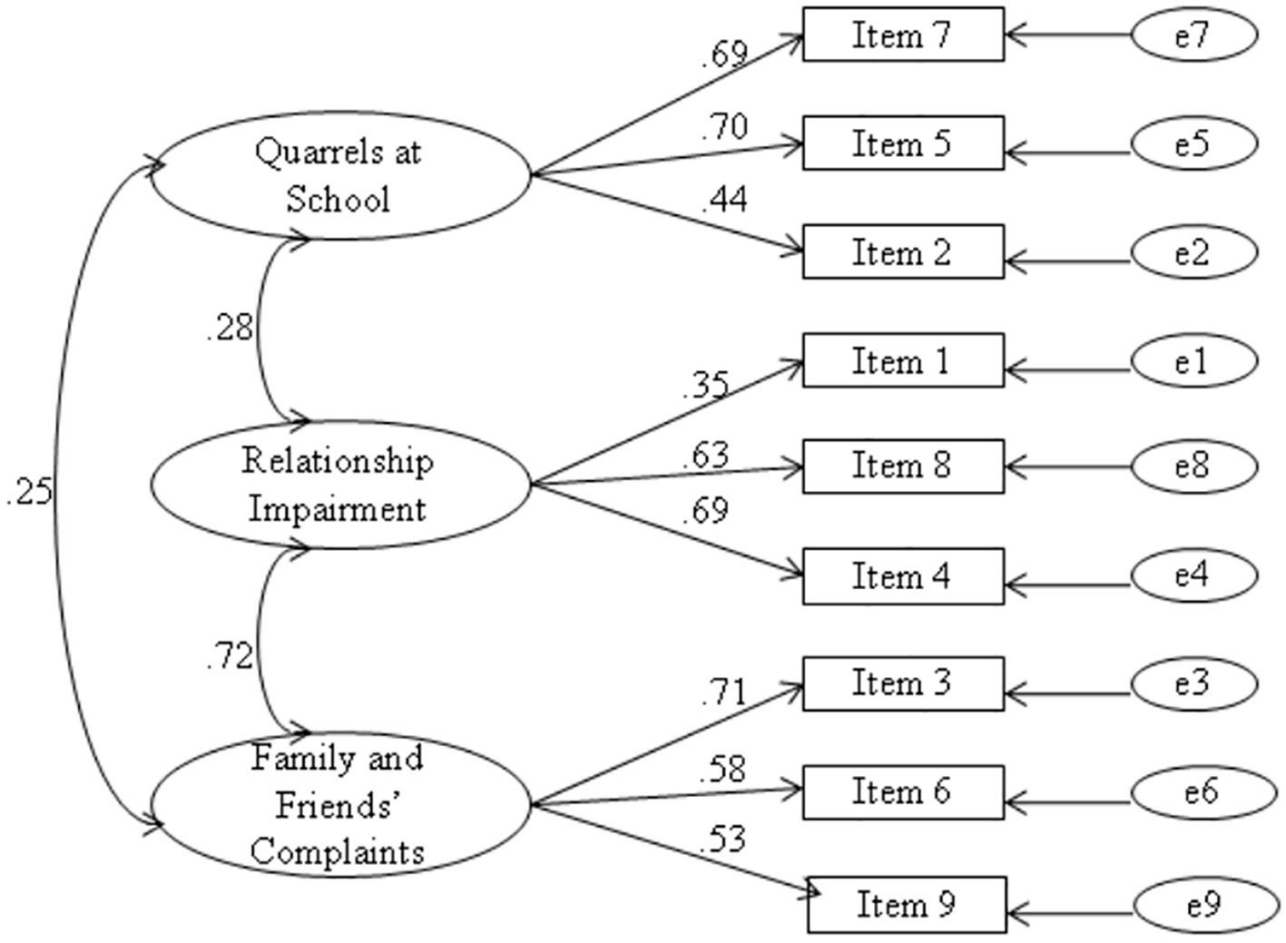
Three-Factor Model, Study-Relationships Conflict Scale, *n* = 308. *Note.* The correlations between errors are not represented.

After that, we performed Confirmatory Factor Analyses (CFAs) on the second sample of students (*n* = 308), which filled the reduced 9-item version of the SRCS. The fit indexes indicate a good fit for the 3-factor model: χ^2^/*df* = 1.99; GFI = 0.96; CFI = 0.95; TLI = 0.92; RMSEA = 0.057, which reach an excellent fit if, following the modification indices suggestions, we allow to correlate two pairs of item errors, namely Error 1 with Error 8 and Error 7 with Error 9: χ^2^/*df* = 1.45; GFI = 0.98; CFI = 0.98; TLI = 0.97; RMSEA = 0.038 (see Figure 1 for the graphical representation of this model).

Then, we calculated the internal reliability and the item-total correlations of the three scales on the total sample (*n* = 598). We found the following Cronbach’s alpha values: QS = .67 (item-total correlations ranging between .76 and .80), RI = .63 (item-total correlations ranging between .74 and .79), and FFC = .64 (item-total correlations ranging between .66 and .82).

Finally, we analyzed the correlation between the three SRCS scales and the five academic indicators: GPA and time spent studying daily and weekly (generally and before exams). The results showed that the GPA correlates with the FFC scale only (*r* = .35, *p* < .001), while the variables related to time investment in study correlate with both RI and FFC, with values ranging between .26 and .35. Finally, QS correlates with Hours a day of study before exams only (*r* = .11, *p* = .008) (see Table 3 for the results).

**Table 3:** Correlations Between the Study-Relationships Conflict Scale (SRCS) Subscales and the Academic Indicators (*n* = 598)

Academic Indicators	SRCS—QS	SRCS—RI	SRCS—FFC
Hours a day of study generally	.05	.35**	.34**
Days a week of study generally	.001	.29**	.29**
Hours a day of study before exams	.11*	.34**	.25**
Days a week of study before exams	.08	.26**	.26**
Grade Point Average	-.04	.07	.35**

*Note.* QS = Quarrels at School; RI = Relationship Impairment; FFC = Family and Friends Complaints.

*p = .008. **p < .001.

## Discussion

This study proposes a new instrument, the Study-Relationships Conflict Scale (SRCS), for the assessment of the negative interference of studying with social (family, friends, and partner) and school (teachers and classmates) relationships, as defined in the present paper.

We developed a pool of 16 items covering these two social domains; more specifically, eight items covered each area. By means of Exploratory and Confirmatory Factor Analyses, we reduced the instrument to nine items and three factors: 1) Quarrels at School—QS, 2) Relationship Impairment—RI, and 3) Family and Friends’ Complaints—FFC. This 9-item version of the test showed an excellent fit. Moreover, all the three SRCS scales have good internal reliability, as they reach the cutoff value of .60 for new scales (Nunnaly & Bernstein, 1994), even if they are composed by three items only. Furthermore, the item-total correlations are good for all the three scales.

Next, we analyzed the correlation between the SRCS scales and some academic indicators: daily and weekly time investment in the study (generally and before exams) and academic performance, or the self-reported Grade Point Average (GPA).

We found that the QS scale weakly correlates with the hours of study per day before exams only (*r* = .11, *p* = .008). The RI scale, instead, has medium values of correlation with all the four variables concerning the time spent studying, but not with the GPA. Finally, the FFC scale shows medium values of correlation with all the academic indicators, including the GPA. Hence, we confirmed all of our hypotheses, as the results showed a correlation between time investment in studying and both RI and FFC, while there is no correlation with QS (even if there is a low correlation between this and hours of study per day before exams). Moreover, we also found that the GPA correlates with FFC only.

These results, taken all together, seem to suggest that the more a student spent his/her time studying and the more he/she experiences relationships’ impairment and family and peers’ complaints about his/her overstudying. Moreover, the higher is the GPA, the higher are the complaints of the family and the peers, but not the quarrels with teachers and classmates and the relationships’ impairment.

The main limit of this study is that the sample is predominantly female, even if it is quite large and representative of many Italian cities and majors of study. Moreover, all the participants are College students; hence, even if the instrument has been designed both in the instruction and in the items in order to be administered to students of various school levels (including pre-adolescents and adolescents), we have not been able to evaluate the psychometric properties of the SRCS on a younger sample. We suggest that future studies aiming to use this scale with pre-adolescents and adolescents should analyze its psychometric properties on these populations before using it.

Beside these limitations, this study has the merit to provide researchers and people working with students (such as psychologists, counselors, or teachers) with an instrument that allows evaluating the negative impact that (over)studying could have on the students’ social and school relationships (RI and FFC scales) and on the school climate (QS scale). Hence, it will allow evaluating how negative (e.g., Studyholism/Study Addiction) and positive (e.g., Study Engagement) study behaviors could affect students’ well-being. Moreover, given its easy and quick way of administration, it could be used in practice to detect if study behaviors that cause an impairment characterize students, and hence to develop preventive interventions aiming to improve their wellbeing.
